# Pyogenic sacroiliitis caused by *Salmonella schwarzengrund* in a young healthy woman: a case report and literature review

**DOI:** 10.1186/s12245-023-00496-y

**Published:** 2023-03-20

**Authors:** Yuki Tokuyama, Hiroyuki Yamada, Ken Shinozuka, Tomoyuki Yunoki, Shigeru Ohtsuru

**Affiliations:** 1 Department of Primary Care and Emergency Medicine, Kyoto University, Kyoto, Japan; 2 Department of Nephrology, Graduate School of Medicine, Kyoto University, Kyoto, Japan

**Keywords:** Non-typhoidal *Salmonella*, Pyogenic sacroiliitis, Psoas abscess

## Abstract

**Background:**

Salmonella species are a leading cause of diarrheal diseases worldwide. Recent epidemiological studies have shown that Salmonella schwarzengrund (S. schwarzengrund) is highly prevalent in various regions. Herein, we report that S. schwarzengrund caused sacroiliac joint (SIJ) infection with septic shock in a young woman, although she was immunocompetent.

**Case presentation:**

A 20-year-old woman presented with left hip pain, accompanied by vasopressor-requiring hypotension. Her imaging examinations showed fluid collection in her SIJ and a small abscess in the left iliac muscle. Later, the blood and aspiration fluid culture and genetic analysis revealed the presence of S. schwarzengrund. We diagnosed sacroiliac joint (SIJ) infection with septic shock caused by S. schwarzengrund. Her condition improved after performing several interventional radiology (IVR) procedures for SIJ abscesses and providing appropriate antibiotic treatment. Finally, she was discharged without any sequelae. Screening tests and genetic analysis about her immunodeficiency did not indicate a congenital disorder.

**Conclusion:**

These clinical courses indicate that S. schwarzengrund could cause the fatal SIJ infection irrespective of the host immunocompetence. Considering the recent increase in the diagnostic rate of S. schwarzengrund, this case emphasized the need to be more cautious about Salmonella species infection.

## Background

*Salmonella* species infection is a major global threat to public health [1, 2]. The most common symptom is gastroenteritis, which is self-limiting [3]. The microorganism does not frequently cause infection at different sites and severe sepsis[3]; however, it could be lethal among immunocompromised hosts such as patients with human immunodeficiency virus (HIV) infection and malignancy and those receiving corticosteroids [4–7]. That is, *Salmonella* species is a common cause of bloodstream infection among individuals living in low-resource areas and may be associated with a high case fatality ratio [8]. Even in developed countries, *Salmonella* infection-related mortality is reported annually [9]. Moreover, recent studies have shown that the diagnostic rate of *Salmonella schwarzengrund* (*S. schwarzengrund*), a specific type of *Salmonella* species, is increasing in both animals and food products worldwide [10–12]. This can then lead to the local outbreak of *Salmonella* infection [13, 14].

Pyogenic sacroiliitis is a relatively rare disease [15]. A recent study showed that it accounts for only 1–2% of all septic arthritis cases [16]. Further, it is associated with low clinical suspicion, vague clinical image, and poorly defined symptom localization [17]. Hence, its diagnosis can be challenging. The main causative pathogens are *Staphylococcus aureus* and *Pseudomonas aeruginosa* because they frequently cause bacteremia [18, 19]. To the best of our knowledge, there are only few case reports on sacroiliitis caused by *S. schwarzengrund*.

Herein, we present a young healthy female patient with *S. schwarzengrund-*related pyogenic sacroiliitis who developed septic shock and review other reported cases.

## Case presentation

**Fig. 1:**
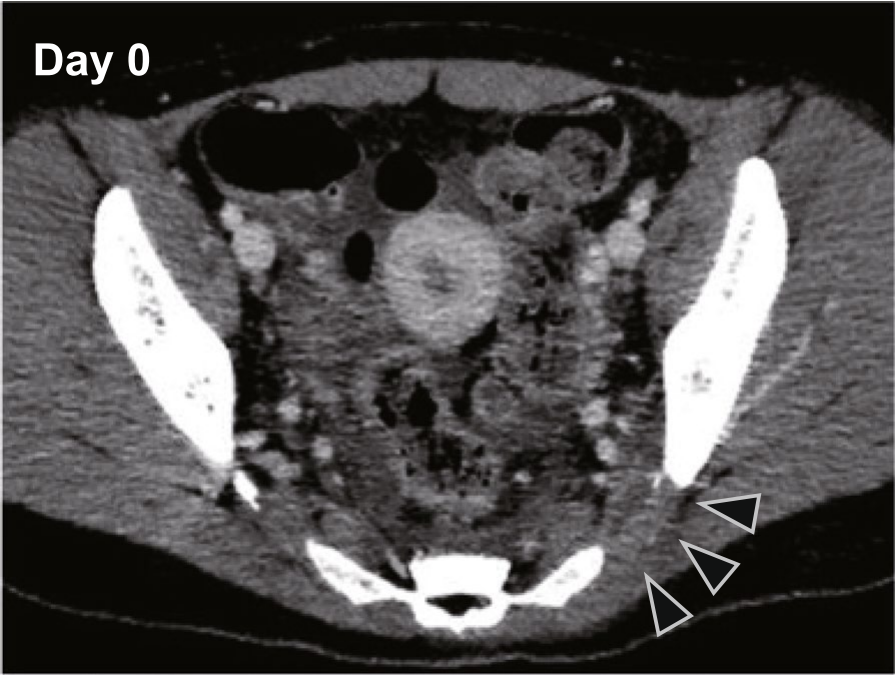
Pelvic computed tomography image upon admission. The arrowhead indicates fluid collection at the left sacroiliac joint

A 20-year-old woman with a history of epilepsy became aware of left hip pain radiating down to the back of her leg for 3 days. The pain progressed gradually and became so severe that she could not move. Thus, she was admitted to the local hospital and received intravenous antibiotics, cefazoline 6 g/day for her possible infection. However, her hip pain worsened, and she developed fever the following day. A pelvic computed tomography scan showed a fluid collection at the left sacroiliac joint (SIJ) and a small abscess in the left iliac muscle (Fig. 1). The next day, her hemodynamic parameters also deteriorated despite the antibiotic treatment, and consciousness disturbance developed. She was transferred to the emergency department of our hospital.

Upon hospital arrival, the patient’s vital signs were as follows: heart rate, 122 beats/min; blood pressure, 95/34 mmHg (norepinephrine 0.16 mcg/kg/min, dobutamine 4.0 mcg/kg/min); body temperature, 36.4 °C; and oxygen saturation while on oxygen therapy at 2 L/min via nasal cannula, 98%. Further, the following are the laboratory test results: C-reactive protein level, 16.8 mg/dL and arterial blood gas lactate level, 4.4 mmol/L (Table 1).

**Table 1:** Blood test findings upon hospital admission

Complete blood counts			Blood chemistry, coagulation		
WBC	(k/mm^3^)	4.76	Blood urea nitrogen	(mg/dL)	23
Neutrophil	(%)	90.6	Creatinine	(mg/dL)	1.44
Lymphocyte	(%)	7.1	Alkaline phosphatase	(U/L)	264
Monocyte	(%)	2.1	Aspartate aminotransferase	(U/L)	1005
Hemoglobin	(g/dL)	10.9	Alanine aminotransferase	(U/L)	403
Hematocrit	(%)	30.8	Total bilirubin	(mg/dL)	1.1
Platelet count	(k/mm^3^)	26	Creatine kinase	(U/L)	2739
Arterial blood gas			Procalcitonin	(ng/mL)	100 <
Lactate	(mmol/L)	4.4	C-reactive protein	(mg/dL)	16.8
Base Excess	(mmol/L)	− 11.2	PT-INR		1.76
HCO_3_	(mmol/L)	17.3	D-dimer	(μg/mL)	40.0 <
CO_2_	(mmHg)	24.3			

Based on the examination results and clinical symptoms, the patient was diagnosed with septic shock caused by SIJ infection. Ultrasonography-guided abscess aspiration was performed to drain the joint fluid and identify the bacterial species. Later, the blood and aspiration fluid culture and genetic analysis revealed the presence of *S. schwarzengrund*.

Broad spectrum antibiotics (meropenem 3 g/day and vancomycin 2 g/day), vasopressors, and oxygen therapy were administered initially. The patient’s hemodynamic and respiratory status gradually improved. After obtaining the culture results, antibiotic treatment was changed to levofloxacin. Ten days after the first drainage, the fever pattern and inflammation markers such as C-reactive protein and erythrocyte sedimentation rate significantly improved.

**Fig. 2:**
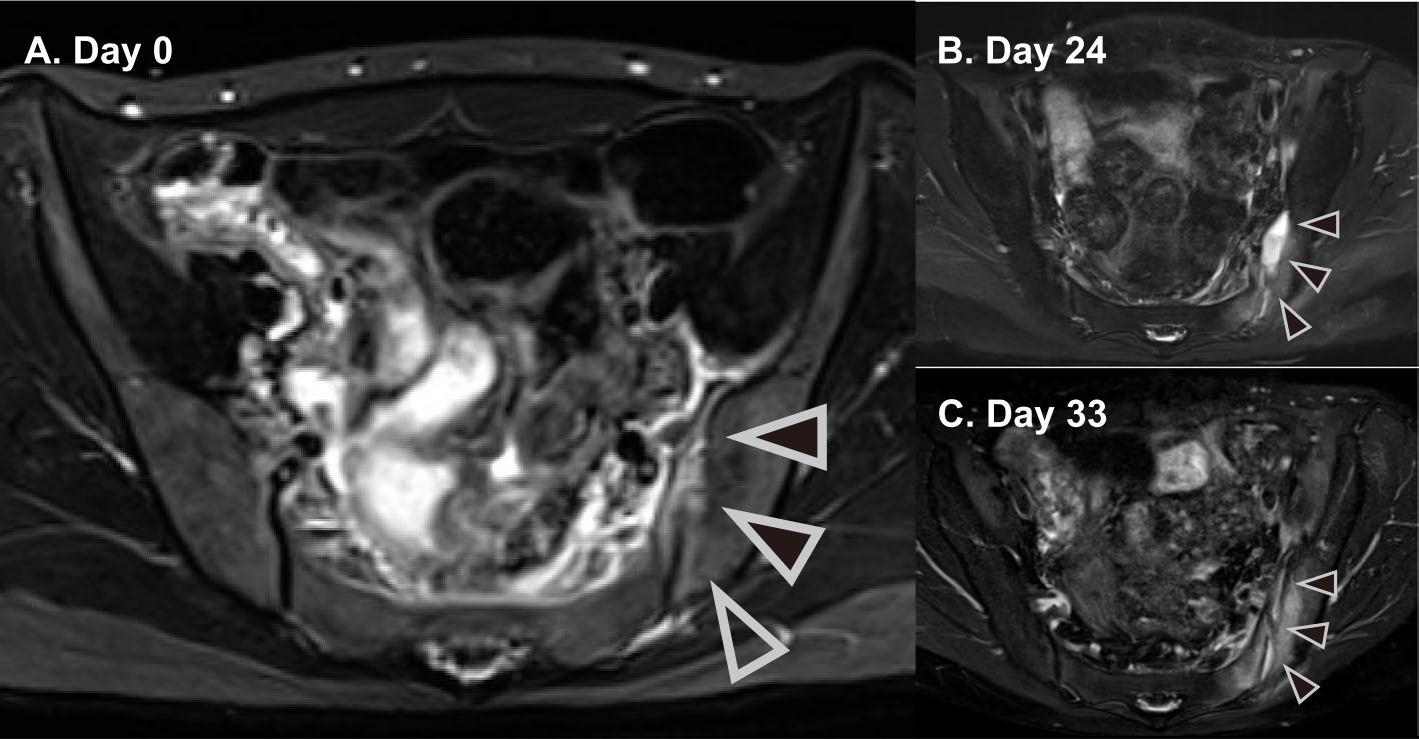
Pelvic magnetic resonance image (**A**) upon admission and days (**B**) 24 and (**C**) 33. The arrowhead indicates fluid collection at the left sacroiliac joint

**Fig. 3:**
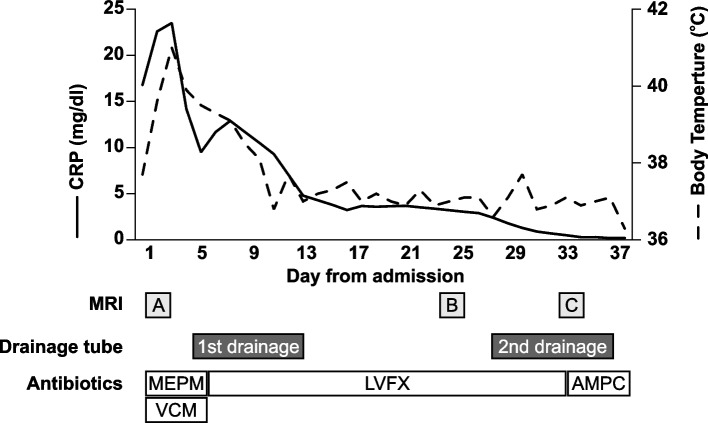
Summary of clinical course

However, after the first drainage tube removal, the patient exhibited persistent fever and inflammation again, and hip magnetic resonance imaging revealed a growing abscess (Figs. 2 and 3). Thus, we performed the second drainage from days 26 to 35. The patient was discharged on day 38 with oral ampicillin treatment for eight more weeks. Upon discharge, she had neither any symptoms nor sequelae (Fig. 3).

To validate the entry route of *S. schwarzengrund*, we interviewed the patient in detail. Except for keeping one dog and two cats, she did not have any specific medical history, such as intravenous drug usage, recent overseas travel, diagnosis of sexually transmitted diseases, or consumption of suspicious food. She did not complain of any preceding gastrointestinal symptoms. The screening test results for immunodeficiency diseases, including HIV infection and autoimmune disorders, were negative. Genetic analysis of congenital immunodeficiency also revealed no significant findings. These results indicated that she did not have any immune system disorders.

## Discussion and conclusion

*Salmonella* species present various kinds of clinical symptoms in human beings. Its common symptom is gastroenteritis, which is self-limiting. Moreover, *Salmonella* causes extraintestinal infections in different organ systems, such as the urinary tract, lung, and central nervous system [3, 20]. Occasionally, this infection can be fatal in immunocompromised hosts.

Our case showed that *S. schwarzengrund* could cause SIJ infection, which is an atypical extraintestinal infection. Additionally, it caused septic shock requiring vasopressors although the patient was young and healthy. These clinical courses indicated *S. schwarzengrund* infection could be a major concern even among immunocompetent patients.

To the best of our knowledge, only two cases of pyogenic sacroiliitis caused by *S. schwarzengrund* have been reported [21, 22]. Generally, extraintestinal *Salmonella* infection occurs in immunocompromised patients, such as elderly individuals and patients with HIV infection [23, 24]. However, as shown in Table 2, *S. schwarzengrund* caused extraintestinal focal infection even in young and healthy individuals. The background characteristics of our patient are consistent with those of patients in previously published reports. Considering the recent outbreak of *Salmonella* infection, it is crucial to be more cautious about the epidemiological status of *S. schwarzengrund* [13, 14].

**Table 2:** Previous cases of *Salmonella schwarzengrund*-related sacroiliitis

Author	Publication	Age/sex	Immunodeficiency	Culture	Treatment	Outcome
Tokuyama et al.	2023	20/F	None	Blood and synovial	Drainage	Improved
Shanahan et al.	1985	19/M	None	Blood	Surgical	Improved
Horgan et al.	1983	18/M	None	Stool and synovial	Surgical	Improved

Another highlight of this case report is the appropriate application of the interventional radiology (IVR) technique. Compared with surgery, the IVR approach is a non-invasive and cost-effective procedure [25, 26]. Nonetheless, it enables adequate drainage of abscesses, similar to our case. Now, SIJ has become a percutaneously accessible site through the development of high-resolution CT and sonography, unlike at the time when the previous reports were published [21, 22]. Therefore, it should be highly desirable to consider its application moving forward.

This study also has certain limitations. First, the removal timing of the drainage tube might be disputable. Second, we could not identify the entry route of *S. schwarzengrund* except via pet food or exposure.

In conclusion, our case report showed that *S. schwarzengrund* could cause SIJ infection, which is fatal regardless of the host’s immunocompetency. Moreover, it emphasized that people should be vigilant and aware of *Salmonella* infections.

## Data Availability

The datasets from this study are available from the corresponding author on request.

## Abbreviations

AMPC: Amoxicillin
HIV: Human immunodeficiency virus
IVR: Interventional radiology
LVFX: Levofloxacin
MEPM: Meropenem
SIJ: Sacroiliac joint
VCM: Vancomycin
